# Pharmacogenetic Testing in an Academic Psychiatric Clinic: A Retrospective Chart Review

**DOI:** 10.3390/jpm11090896

**Published:** 2021-09-08

**Authors:** Lisa Brown, James Li, Naryan Katel, Kunbo Yu, Evangelia Fatourou, Brett Himmler, Angelos Halaris

**Affiliations:** 1 Myriad Neuroscience, Salt Lake City, UT 84108, USA; jimli2323@gmail.com (J.L.); naryan.katel@gmail.com (N.K.); yukunbo51@gmail.com (K.Y.); b.t.himmler@gmail.com (B.H.); 2 Department of Psychiatry and Behavioral Neuroscience, Stritch School of Medicine, Loyola University Chicago and Loyola University Medical Center, Maywood, IL 60660, USA; fatouroe@nychhc.org (E.F.); ahalaris@luc.edu (A.H.)

**Keywords:** pharmacogenomics, depression

## Abstract

Pharmacogenomic (PGx) testing is being increasingly recognized by clinicians as an essential tool to guide medication decisions for treatment of psychiatric illnesses. Extensive implementation of PGx testing, however, varies by setting and location. In this retrospective study, we reviewed charts from 592 patients diagnosed with a psychiatric disorder at the Loyola University Medical Center, for whom PGx testing was performed. Information collected included demographics at the time of testing, psychiatric diagnosis, medical and psychiatric history and medications prior and after PGx testing. Of the 592 charts analyzed, the most common primary diagnoses were depression (52%) and anxiety (12%). Prior to PGx testing, 72% of patients were prescribed three or more medications, whereas, after testing, only 44% were prescribed three or more medications included in the test panel (*p* < 0.0001). The most common clinical consideration on the PGx reports was recommendation to reduce dosages (33%). After PGx testing, the proportion of patients taking incongruent medications decreased from 26% to 19% and that of patients taking congruent medications increased from 74% to 81% (*p* = 0.006). The results from this retrospective data analysis demonstrated a reduction in polypharmacy and an increase in recommendation-congruent medication prescribing resulting from implementation of PGx testing.

## 1. Introduction

Major depressive disorder (MDD) has a lifetime prevalence of 20.6% and only half of patients respond to their first antidepressant, resulting in a potential lifetime of trial and error [1,2]. Studies such as STAR*D have shown that the likelihood of remission from depression only decreases with each additional medication trial and the risk of adverse events increases with each trial [2]. This trial and error approach also increases the development of treatment resistant depression (TRD), which incurs significantly greater healthcare utilization and costs compared to non-TRD MDD, while prolonging pain and suffering and overall dysfunctionality [3]. Therefore, there is an urgent need to significantly improve the rates of response and remission, this being the gold standard of care. Novel tools are needed to personalize treatment choices therefore improving outcomes and decreasing side effects [4].

One tool that clinicians have utilized over the last decade to help personalize treatment decisions is pharmacogenomic (PGx) testing [5]. PGx testing utilizes genomic sequencing to determine pharmacokinetic, immune-related, and pharmacodynamic effects of variants in specific genes that can affect an individual’s response to medications. The greatest evidence for the value of PGx testing in psychiatry has been demonstrated in individuals with depression who have failed at least one medication [6,7]. Two meta-analyses showed that patients whose treatment was guided by PGx testing, resulted in improved remission of symptoms compared to treatment as usual [6,7]. Therefore, PGx testing may be a valuable tool for clinicians in guiding and personalizing medication decisions in conjunction with clinical expertise [8]. Previous studies have shown clinical validity of the severity of gene–drug interactions (GDI) in guiding medication decisions and improving outcomes [4,9,10]. A meta-analysis for one individual commercial test also showed significantly greater outcomes in individuals with depression whose treatment was guided by PGx testing, compared to treatment as usual [11].

While blinded, randomized controlled trials (RCTs) are the gold standard in evaluating a new intervention, it is crucial to also understand implementation and utilization of PGx testing in a real-world setting [12,13]. Therefore, we sought to perform this retrospective quality improvement (QI) project to understand how PGx testing is used in a psychiatric clinic at an academic medical center. 

## 2. Materials and Methods

The study was approved by the Institutional Review Board (IRB) of the Loyola University Medical Center. The study was supported by Myriad Neuroscience and results were compared to those from QI projects at other sites with the same protocol. Charts were identified as any individual in the Ambulatory Service of the Department of Psychiatry who received PGx testing between January 2010 and June 2019. All patient information was deidentified prior to data analysis. Inclusion criteria for chart review included the following at the time of PGx testing: diagnosis of a psychiatric disorder, age greater than eight years old and availability of relevant clinical information. A total of 600 charts were reviewed. Eight patients were excluded due to insufficient data and 592 were deemed eligible for inclusion in this analysis. The following information was collected, if available: demographics (e.g., gender, age, race/ethnicity), diagnosis of a psychiatric disorder, medical, neurological and psychiatric history, all psychotropic and concomitant medications (within 1 year prior to, at the time of and up to 2 years following the PGx test date), investigator’s assessment of medication efficacy (e.g., CGI, PHQ-9), physical examination and vital signs and all adverse events (AEs) (within 1 year prior to, at the time of and up to 1 years following PGx testing).

The specific PGx test used in this population was the GeneSight test (Myriad Genetics, Mason, OH, USA). This PGx test relies on a combinatorial algorithm evaluating multiple gene effects on each individual psychotropic medication. The algorithm ultimately “bins” medications based on severity of gene–drug interactions (GDI) into three color-coded categories: green for “no known GDI,” yellow for “moderate GDI,” or red for “significant GDI”.

A medication decision is defined as “congruent” if the patient was prescribed a medication categorized as “green” or “yellow” within the one-year period of interest. The medication decision is considered “incongruent,” if a “red” category medication was prescribed during this period. For proportions of congruent and incongruent analysis, the McNemar test was used to compare the proportion of before and after PGx testing. Differences are considered statistically significant if *p*-value < 0.05 (2-sided). SAS 9.4 was used for all analyses.

## 3. Results

### 3.1. Demographics

**Figure 1:**
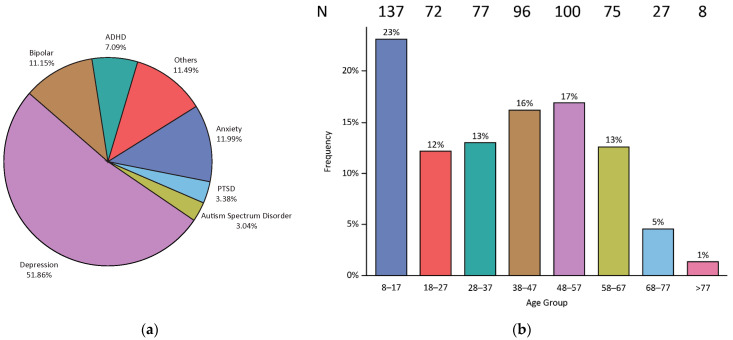
Patient diagnoses and ages. (**a**) A majority of individuals receiving PGx testing had a primary diagnosis of MDD or anxiety disorder followed by other and bipolar disorders. (**b**) Patients ranged in age from 8 to greater than 77 years, but a majority were between the ages of 8 and 17. ADHD: Attention Deficit Hyperactivity Disorder PTSD: Post-Traumatic Stress Disorder.

Of 600 charts reviewed, 592 met inclusion criteria for this analysis (Table 1). Of the 592 charts analyzed, depressive and anxiety disorders were the most common primary diagnoses at 52% and 12%, respectively (Figure 1a). Patients ranged from 8 to 88 years in age and the median age was 39 (Figure 1b). Of the population analyzed, 65% were female and 63% were Caucasian (Table 1). 

### 3.2. Congruency

In the congruency analysis, medications were included if they had been prescribed between one year before the PGx testing date and one year after the PGx testing date plus 45 days. The 45-day timeframe was deemed sufficient for the clinician to make appropriate medication changes based on the PGx testing. Congruency for 125 patients could not be established as 47 patients did not take any medications on the PGx panel prior to PGx testing and 78 patients did not have any record of medication taken within one year after the PGx testing date. 

**Figure 2:**
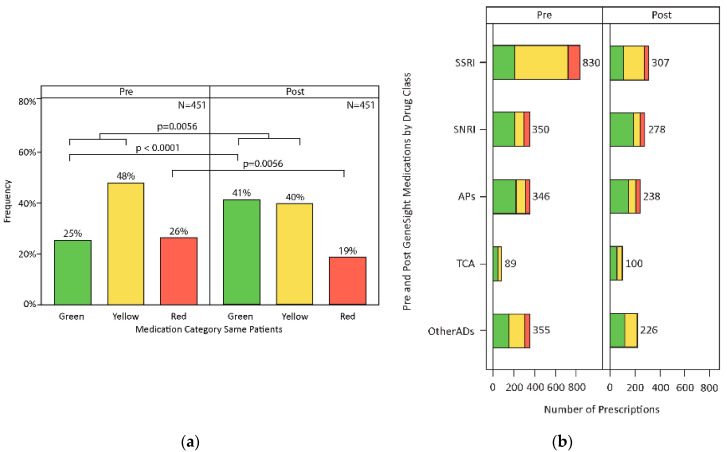

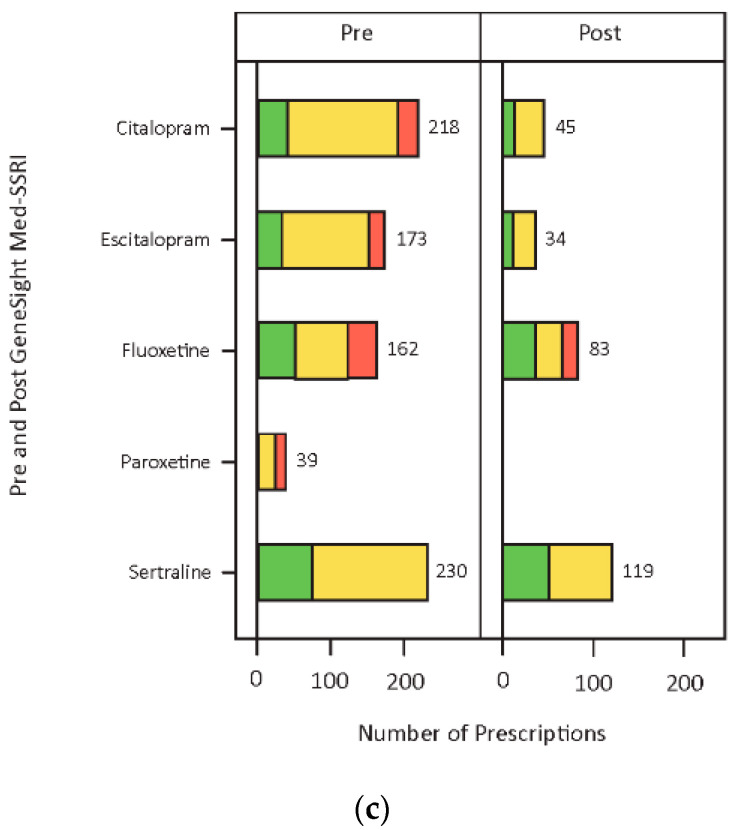
Medication congruency pre- and post-PGx testing. (**a**) There was a significant reduction in individuals taking incongruent medications post-PGx testing. (**b**) The greatest reduction in medication class was that of SSRIs. (**c**) Es/Citalopram had the greatest reduction in prescriptions post-PGx testing.

For the post-PGx period, the proportion of patients taking red category medications decreased from 26% to 19% and that of patients taking green or yellow category medications increased from 73% to 81% (Figure 2a; *n* = 451, *p* = 0.0056). In the total Myriad QI cohort (including other sites, *n* = 1984), the proportion of patients taking red category medications decreased from 26% to 16% and patients taking green or yellow medications increased from 74% to 84% (Figure S1; *p* < 0.0001). 

When the analysis was focused on 451 patients who took green, yellow, or red medications in the year before the PGx testing, 91% of the patients who took green category medication continued taking green or yellow medications in the year following PGx testing. Of patients who took yellow medications before the PGx test, 90% took green or yellow medications after PGx testing. Of patients who took red medications before the PGx testing, 56% switched to taking green or yellow medications after PGx testing (*p*-value = 0.0004).

### 3.3. Medication Changes

**Figure 3:**
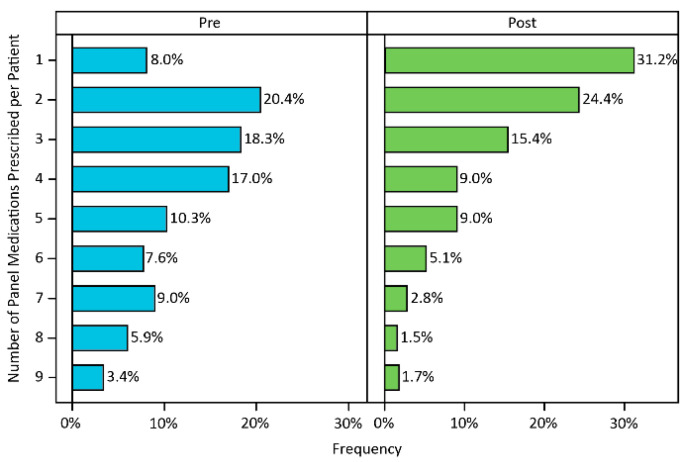
Number of prescribed psychotropic medications pre- and post-PGx testing. Prior to PGx testing, a majority of patients were taking four or more psychotropic medications, whereas, post-PGx testing, this number dropped to a majority taking two or fewer psychotropic medications.

Prior to PGx testing, the most prescribed medications were sertraline, citalopram and clonazepam (Table S1). After PGx testing, the most prescribed medications were desvenlafaxine, sertraline, clonazepam and hydrocodone. SSRIs were the most frequently prescribed medication class before and after PGx (Figure 2b,c; Table S1 and Figure S2). Considering the number of panel medications patients were prescribed, 53% of patients were prescribed four or more panel medications pre-PGx testing, while only 29% were prescribed four or more panel medications post-PGx test (Figure 3; *p* < 0.0001). Similarly, considering overall polypharmacy, 70% of patients pre-PGx testing were prescribed four or more medications (panel medication or otherwise), while 41% of patients were prescribed a similar number of medications post-PGx testing. Similar to the total Myriad QI cohort, the highest number of patients were taking one or two medications post-testing (Figure S3; *p* < 0.0001).

### 3.4. Adverse Events

Adverse events (AE) were not systematically documented for patients prior/post to PGx testing; therefore, AE analysis was not performed.

### 3.5. Clinical Considerations 

**Figure 4:**
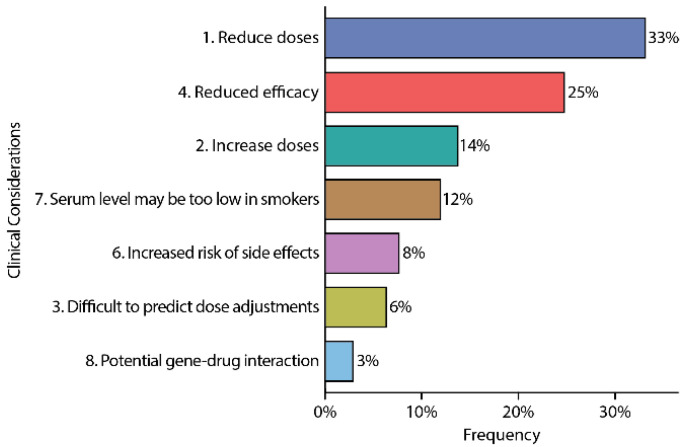
Frequency of clinical considerations. Of the types of gene–drug interactions, the most common was a recommendation to reduce the dose due to decreased metabolism. The second most common was related to reduced efficacy due to metabolism or pharmacodynamic gene profile. A total of 3% had an important FDA label GDI.

Among all the panel medications that patients took during the study period, seven types of explanatory footnotes appeared 2078 times for 47 different medications (Figure 4). The footnotes on this specific PGx test refer to the clinical implications of the identified GDI. Of all the footnotes, 33% were footnotes #1—“Serum level may be too high, lower doses may be required”—indicating that one third of the medications/population had at least one GDI predicting higher than expected blood levels and recommended to use lower dosages. The second most common footnote was #4—“Reduced efficacy”—indicating that one quarter of the medications were expected to have lower efficacy for these patients. In the cohort, medications with FDA verbiage for gene–drug interactions were identified in 3% of medications/patients.

## 4. Discussion

The goal of this quality improvement (QI) study was to understand how pharmacogenomic (PGx) testing utilization and implementation at an academic medical center changed prescribing practices of clinicians for individuals with psychiatric disorders. The study of almost 600 patients showed that clinicians were more likely to prescribe recommendation-congruent medications after PGx testing, or medications with fewer gene–drug interactions (GDI). The approach taken by performing this PGx test to establish medication congruency with this commercial test is valuable and validates previous studies reporting that PGx testing can guide clinicians to prescribe medications with fewer GDIs, reduce trial and error and ultimately achieve higher response and remission rates [9,14,15]. It is important to emphasize that data from studies are at the population level; outcomes studies show that replacing “red”-classified medications with “green”- or “yellow”-classified medications for a given patient results in better outcomes. However, on an individual level, a “red”-classified medication may still be prescribed, if, in the opinion of the prescriber, this is an appropriate choice for reasons other than PGx GDI information. In such a case, it is paramount to heed the cautionary footnote explaining why this particular agent has been classified as “red” for the particular patient and observe the clinical implication note [16]. Therefore, it is important to use PGx testing in conjunction with clinical expertise and the presenting clinical features of the patient receiving treatment [17]. As noted in the present study cohort, where the prescriptions of “red”-classified medications were reduced post-testing, but not totally avoided, the clinician opted to keep the patient on a “red” medication, ostensibly for other compelling clinical reasons. Since this is a retrospective, rather than prospective study, clinicians were not required to use the PGx information in making decisions and this factor may have diluted the overall adherence to post-testing congruency. 

Of note, the percentage (26%) of patients initially taking genetically incongruent medications was higher than the 20.6% incongruency in a recently published large study using this PGx test in patients with depressive disorder [4]. This indicates that the Loyola patient population was enriched in patients taking genetically suboptimal medications. A plausible explanation for this observation could be the fact that insurers mandate medication choices based on their formularies rather than the best judgement of the treating prescriber.

In previous PGx studies, patients whose clinician did not follow PGx guidance (patients remained on red medications) had a significant increase in healthcare visits, medical absence days, disability claims and spent USD 5188 more on healthcare than patients who followed PGx guidance (on green or yellow category medications) [18]. In another study looking at medication cost savings, patients whose treatment was congruent with the PGx test resulted in 1-year medication savings of USD 1035 [19]. Interestingly, a post-hoc analysis of the medication trial also found that patients receiving psychotropic prescriptions from a primary care provider saved USD 3988.00 per year in medication costs, suggesting that improvement in psychiatric disease also resulted in savings for non-CNS medications [20].

While phenotype frequencies differ among ethnicities, when comparing the phenotype frequencies of the Loyola QI population to the general world population, the Loyola cohort found an enrichment in CYP2D6 UMs with a frequency of 0.05 (Table 2) [21]. Of note, the Loyola sample refers to *1/*17 as NM, whereas the Clinical Pharmacogenetics Implementation Consortium (CPIC) and PharmVar reference refers to *1/*17 as UM.

Probably, the most striking finding from this study is the reduction of polypharmacy of psychiatric medications from pre- to post-PGx testing. This demonstrates that PGx testing may decrease polypharmacy, which may also reduce the number of drug–drug interactions, as well as cost to healthcare systems and patients [18]. Reducing polypharmacy also reduces the risk for adverse reactions, a top reason for emergency room visits. Last but certainly not least, reversing TRD by means of PGx testing enables the patient who has suffered from repeated medication failures to regain a reasonable degree of functionality, if not outright full remission of symptoms.

### Limitations

The primary limitation of this study is the lack of quantified clinical outcomes and control subjects. A prospective case-controlled group would have allowed a more direct comparison between the utilization of PGx testing versus treatment as usual. Therefore, no definitive conclusions about efficacy of PGx testing can be drawn from this retrospective study. Another limitation is that all data were extracted by hand from medical records and this may have resulted in missing data due to lack of tracking in the record or human error in recording. However, other techniques to extract data, such as natural language processing, are not without their limitations either. The present data were also analyzed based on the primary diagnosis rather than secondary or other comorbidities that were not taken into account in the analyses. Of note, because this study was an observational retrospective study, treating providers were not required to use the PGx test in treatment decision-making, therefore potentially diluting the effect of the intervention. We were also unable to confirm that the PGx report was even considered by the treating clinician. Finally, a limitation of this study is that medications beyond psychotropics for comorbidities were not collected. Concomitant medications and comorbidities can affect drug–drug and drug–gene–drug interactions that may affect how the body responds to medication in conjunction with PGx information.

## 5. Conclusions

In this retrospective study, we sought to investigate the natural implementation and utilization of PGx testing in an academic psychiatric clinic setting. We found that individuals who had PGx testing were prescribed medications with fewer GDIs post-PGx testing (Figure 2a). We also observed a marked decrease in polypharmacy post-PGx testing (Figure 3). PGx testing can be a valuable tool to improve treatment decisions and, potentially, beneficial patient outcomes in psychiatric populations.

## Figures and Tables

**Table 1:** PGx cohort demographics.

Characteristic	Mean or Frequency	Standard Error or Percent
Age	38.12	0.8
Age group		
8–17	137	23.10%
18–27	72	12.20%
28–37	77	13.00%
38–47	96	16.20%
48–57	100	16.90%
58–67	75	12.70%
68–77	27	4.60%
>77	8	1.40%
**Number of panel-tested psychotropic medications prescribed prior to PGx testing **	4.76	0.13
0 or missing	15	2.50%
1	42	7.10%
2	107	18.10%
3+	428	72.30%
**Gender**		
Male	205	34.60%
Female	385	65.00%
Not provided	2	0.30%
**Race**		
White	374	63.20%
Other	63	10.60%
Not provided or missing	155	26.20%
**Ethnicity**		
Hispanic/Latino	80	13.50%
Not Hispanic/Latino	498	84.10%
Not provided	14	2.40%
**Current smoker**		
Yes	95	16.10%
No	485	81.90%
Not provided	12	2.00%
**Primary Psychiatric Diagnoses**		
Major depressive disorder	307	51.90%
Post-traumatic stress disorder	20	3.40%
Attention deficit disorder	42	7.10%
Anxiety	71	12.00%
Autism spectrum disorder	18	3.00%
Bipolar disorder	66	11.20%
Others	68	11.50%

**Table 2:** Population phenotype frequencies of the world and Loyola sample.

Gene	Population	PM	IM	NM	UM
*CYP2D6*	World	0.049	0.287	0.644	0.028
	Loyola	0.01	0.24	0.59	0.05
*CYP2C19*	World	0.042	0.290	0.386	0.287
	Loyola	0.03	0.19	0.73	0.04

PM, poor metabolizer; IM, intermediate metabolizer; NM, normal metabolizer; UM, ultrarapid metabolizer.

## Data Availability

The data that supports the findings of this study are not publicly available. Deidentified data presented in this study may be made available on request from the corresponding author.

## Supplementary Materials

The following are available online at https://www.mdpi.com/article/10.3390/jpm11090896/s1, Figure S1: Myriad congruency of medications pre- and post-PGx testing, Figure S2: Loyola medication congruency pre- and post-PGx testing, Figure S3: Number of prescribed medication pre- and post-PGx testing, Figure S4: Phenotype frequencies between Loyola QI and Myriad QI cohorts, Table S1: Medications pre- and post-PGx testing.
