# Supplementary material for: Pharmacogenetic Testing in an Academic Psychiatric Clinic: A Retrospective Chart Review
Source: J Pers Med. 2021 Sep 8;11(9):896. doi: 10.3390/jpm11090896 (PMC8470368; doi:10.3390/jpm11090896)
Supplement: Supplementary file 1 [file jpm-11-00896-s001.zip › jpm-1358257-SI.pdf]

Supplemental Table S1. Medications pre- and post-PGx testing

	Pre-PGx Testing				Post-PGx Testing			
**Medication**	**Green**	**Yellow**	**Red**	**Total**	**Green**	**Yellow**	**Red**	**Total**
alprazolam	87	10	.	97	42	.	.	42
amitriptyline	33	15	10	58	27	22	.	49
aripiprazole	51	31	.	82	27	16	12	55
atomoxetine	.	.	.	.	18	.	.	18
bupropion	47	45	17	109	32	30	.	62
buspirone	22	.	.	22	22	.	.	22
citalopram	41	152	25	218	13	32	.	45
clonazepam	186	26	.	212	91	10	.	101
clonidine	23	19	19	61	12	.	.	12
cyclobenzaprine	15	45	.	60	23	.	.	23
desvenlafaxine	112	.	.	112	130	.	.	130
dexmethylphenidate	12	.	.	12	11	.	.	11
dextroamphetamine	11	.	.	11	.	.	.	.
diazepam	18	.	.	18	13	15	.	28
duloxetine	23	35	44	102	18	22	29	69
escitalopram	34	118	21	173	11	23	.	34
fentanyl	37	14	.	51	25	.	.	25
fluoxetine	51	71	40	162	34	32	17	83
guanfacine	23	11	.	34	18	.	.	18
hydrocodone	102	39	26	167	61	22	18	101
hydromorphone	29	.	.	29	.	.	.	.
ibuprofen	30	13	.	43	10	.	.	10
lamotrigine	63	.	16	79	43	.	.	43
lorazepam	61	20	.	81	28	15	.	43
methylphenidate	46	36	.	82	41	23	.	64
mirtazapine	13	41	33	87	.	25	13	38
morphine	26	.	.	26	15	.	.	15
naproxen	28	.	.	28	11	.	.	11
nortriptyline	12	.	.	12	30	.	.	30
olanzapine	15	16	17	48	10	17	17	44
oxcarbazepine	13	.	.	13	14	.	.	14
oxycodone	26	.	.	26	11	.	.	11
paroxetine	.	22	18	39	.	.	.	.
quetiapine	112	24	.	136	83	.	.	83
risperidone	24	15	.	39	12	11	.	23
sertraline	75	155	.	230	50	69	.	119
tramadol	29	23	12	64	17	.	.	17
trazodone	68	56	.	124	47	30	.	77
venlafaxine	70	49	17	136	42	29	.	71
vortioxetine	23	.	.	23	35	.	.	35
ziprasidone	10	.	.	10	10	.	.	10

zolpidem	32	.	.	32	18	.	.	18
**Total**	1733	1101	315	**3148**	1155	443	106	**1704**

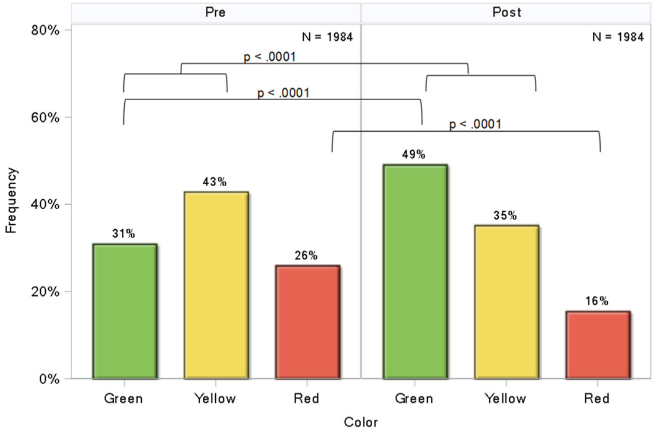

Supplemental Figure S1. Myriad congruency of medications pre- and post-PGx testing.

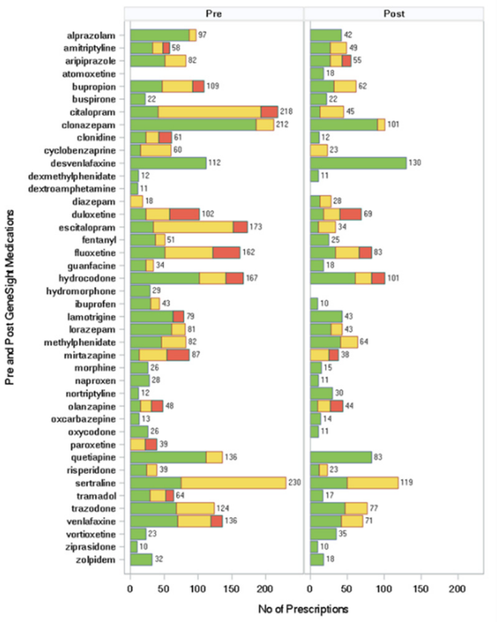

Supplemental Figure S2. Loyola medication congruency pre- and post-PGx testing.

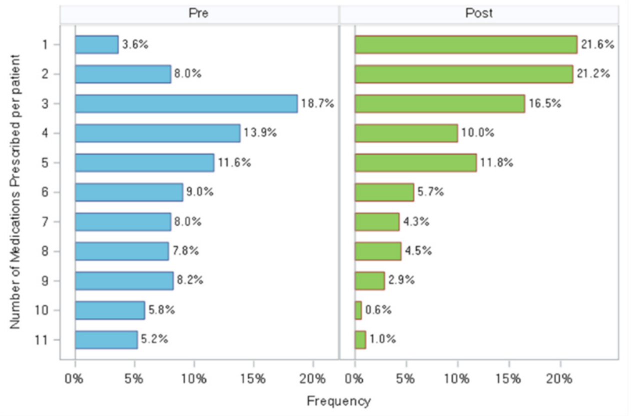

Supplemental Figure S3. Number of prescribed medication pre- and post-PGx testing.

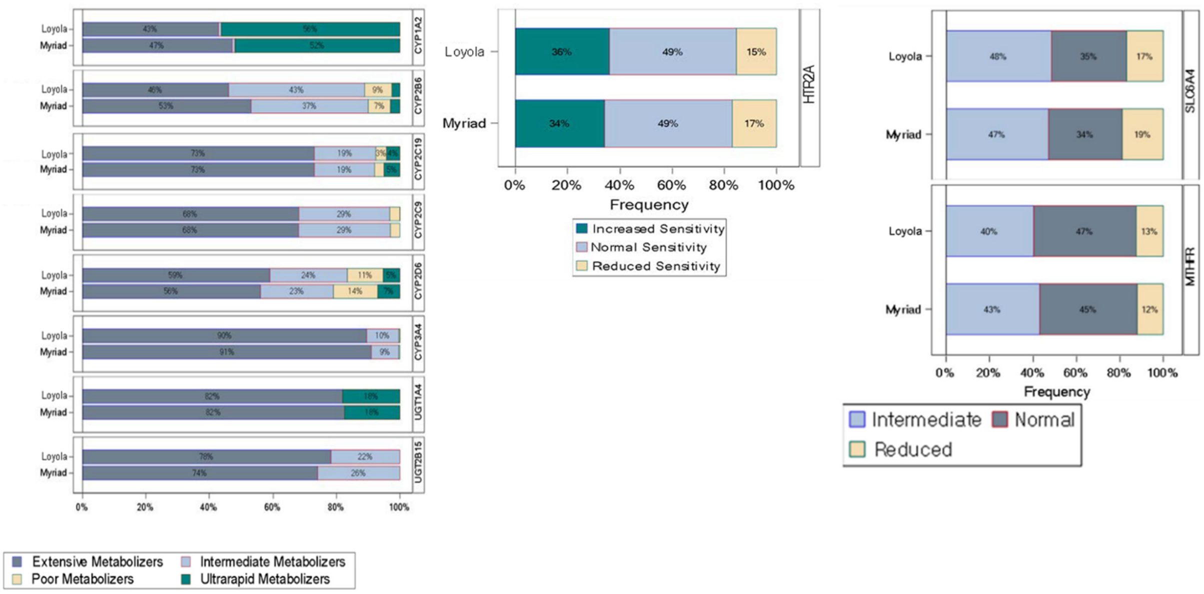

(**a**) (**b**) (**c**)

Supplemental Figure S4. Phenotype frequencies between Loyola QI and Myriad QI cohorts.
